# Posterior Reversible Encephalopathy Syndrome in a Patient with Multiple System Atrophy and Multiple Myeloma

**DOI:** 10.1002/mdc3.70508

**Published:** 2026-01-08

**Authors:** Jackson Mitzner, Jenny Linnoila, Yanal Shaheen, Abby L. Olsen

**Affiliations:** ^1^ Department of Neurology University of Pittsburgh, UPMC Pittsburgh Pennsylvania USA

**Keywords:** multiple system atrophy

Posterior Reversible Encephalopathy Syndrome (PRES) is a neurological disorder characterized by vasogenic edema, typically associated with acute hypertension, renal dysfunction, and/or exposure to cytotoxic medications. Multiple system atrophy (MSA) is a neurodegenerative movement disorder marked by autonomic dysfunction, parkinsonism, and cerebellar dysfunction. PRES has been reported previously in the context of MSA, but all reported cases involved patients taking medications such as midodrine or fludrocortisone, which can exacerbate supine hypertension.^1^, ^2^, ^3^ Here we describe a patient presenting with PRES, eventually leading to a diagnosis of MSA‐C. She also had a history of multiple myeloma recently in remission and was maintained on lenalidomide, an immunomodulatory therapy.

## Case Report

**Figure 1:**
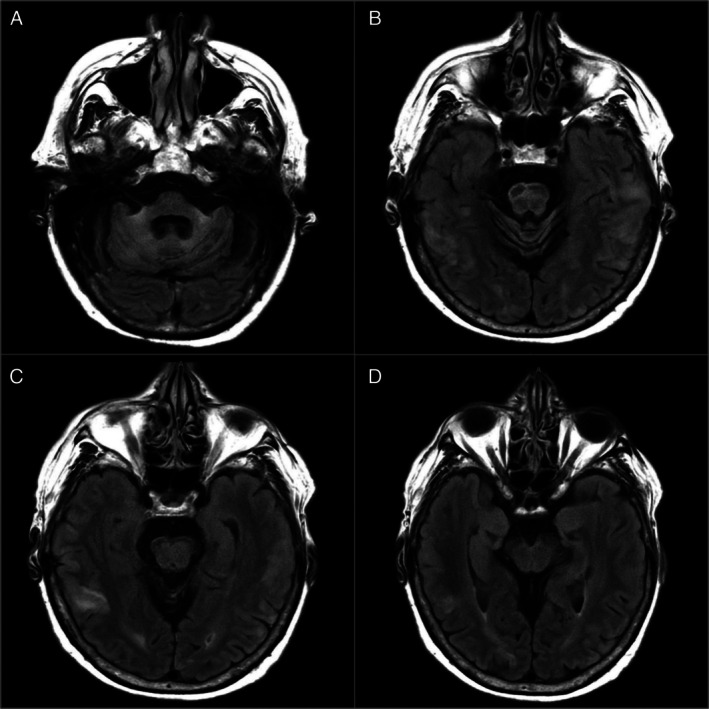
T2/FLAIR images obtained on presentation to the emergency department with acute hypertension and confusion. Note significant gliosis of the middle cerebellar peduncle (A) and prominent pontine cruciform hyperintensity or “hot cross buns sign” (B). Additionally, there are several bilateral temporal and occipital lesions (B–D), predominantly posterior, highly suggestive of posterior reversible encephalopathy syndrome (PRES), most prominent in the R temporal lobe (C).

A 78‐year‐old right‐handed woman presented to the emergency department with acute onset headache and confusion. She had a history of multiple myeloma and had undergone bone marrow transplant 3 years prior. She was in remission at the time of admission, and on maintenance therapy with lenalidomide. Other home medications included acyclovir, ascorbic acid, aspirin, calcium‐vitamin D, cyanocobalamin, a multivitamin, and timolol ophthalmic eye drops. She was retired from her job as an administrator. Her family had noticed several days of worsening lethargy, and elevated blood pressure to the 170 s systolic, despite no history of hypertension. She developed a severe headache and progressive confusion, which prompted her family to take her to the hospital. On arrival her blood pressure was 204/96. She was stuporous and minimally responsive. MRI brain revealed bitemporal and occipital T2/FLAIR hyperintensities, suspicious for PRES (Fig. 1). Additional workup, including CSF testing and continuous EEG monitoring, was unremarkable. With acute blood pressure control, her mental status gradually returned to baseline over the next week.

**Figure 2:**
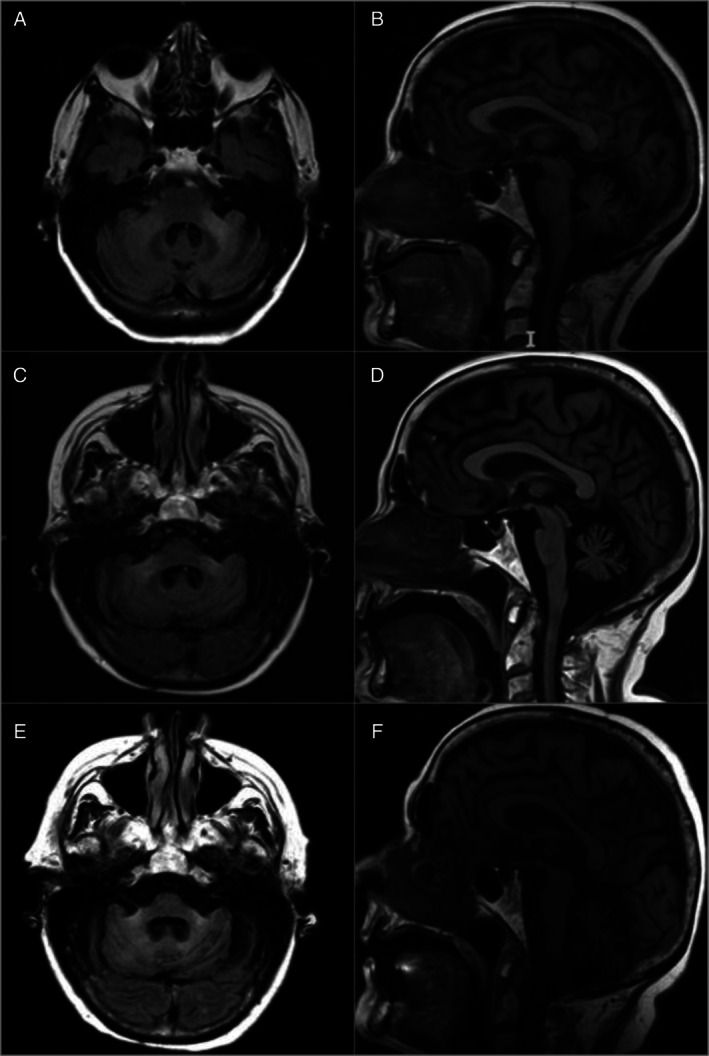
Serial MRI images taken over several years preceding the patient's acute presentation to the emergency room (ED). Note progressive atrophy and gliosis of the cerebellar peduncles and pons. (A, B) Four years prior to presentation. (C, D) Two years prior to presentation. (E, F) MRI from day of presentation to the ED.

In addition to the T2/FLAIR changes concerning for PRES, her MRI was also remarkable for cruciform T2 hyperintensity in the pons (“hot cross buns sign”), as well as marked gliosis and atrophy of the cerebellum and middle cerebellar peduncles (Figure 1 and 2). The patient had recently sought neurologic evaluation for several signs of progressive cerebellar degeneration, including hypometric saccades, ataxic dysarthria, dysmetria, dysdiadochokinesia, and wide‐based gait, which had appeared and slowly progressed since her bone marrow transplant. Outpatient work‐up was underway for a possible paraneoplastic disorder, given her history of multiple myeloma. A paraneoplastic panel noted positive IgG binding to monkey cerebellar substrate, but identified no known neural autoantibodies. Retrospective review of prior brain MRIs revealed progressive pontine and cerebellar atrophy (Fig. 2).

Concurrently with the cerebellar signs, she had developed chronic urinary retention causing incontinence, again without clear etiology. She did not have a history of documented orthostatic hypotension, but she did endorse symptoms of orthostasis while standing. After the episode of PRES, she was referred to a movement disorders specialist. Given her progressive cerebellar dysfunction, unexplained autonomic dysfunction, and characteristic imaging findings, the diagnosis of MSA—cerebellar subtype (MSA‐C) was clinically established, using the 2022 Movement Disorders Society criteria. Specifically, she fulfilled the core clinical features due to unexplained urinary urge incontinence and a cerebellar syndrome with gait ataxia, limb ataxia, cerebellar dysarthria, and oculomotor features. She fulfilled supportive criteria including rapid progression with 3 years of motor onset, moderate to severe postural instability within 3 years of motor onset, severe speech impairment within 3 years of motor onset, and inspiratory sighs. She fulfilled MRI criteria, including atrophy of the pons and middle cerebellar peduncle, hot cross bun sign, and increased diffusivity of the putamen. Her neurological exam revealed cerebellar findings consistent with this diagnosis (Video 1 and 2).

**Video 1:** The patient's exam, filmed after her recovery from posterior reversible encephalopathy syndrome (PRES), reveals prominent cerebellar features, including cerebellar dysarthria (scanning speech).

**Video 2:** The patient's exam demonstrates dysmetria on finger‐nose‐finger and heel‐to‐shin testing, and a wide stance. Her gait was markedly ataxic, and she was unable to walk without 2‐person assistance. There is mild bradykinesia, slightly worse on the left than the right, and rigidity (not demonstrated).

## Discussion

PRES is typically associated with hypertensive encephalopathy, renal dysfunction, and/or exposure to immunosuppressive or cytotoxic drugs. Our patient was taking maintenance lenalidomide, which may be associated with PRES, though the available literature is limited.^4^, ^5^ In the context of MSA, prior cases of PRES have been reported in patients taking medications that elevate blood pressure.^1^, ^2^, ^3^ Our patient was not on these medications, raising the possibilities of (1) autonomic dysregulation from MSA, (2) lenalidomide treatment, or a combination of the two as potential etiologies of PRES in this case.

Autonomic failure, such as that seen in MSA, can lead to severe blood‐pressure fluctuations, with supine hypertension resulting from an impaired baroreceptor reflex and orthostatic hypotension due to sympathetic failure.^6^, ^7^, ^8^ The pathogenesis of PRES in this setting likely involves a failure of cerebral autoregulation in response to these fluctuations. PRES has also been rarely associated with other disorders of autonomic instability in the absence of chronic hypertension, such as Guillain‐Barré Syndrome (GBS) or spinal cord injury.^9^, ^10^ These associations indicate that transient severe hypertension may exceed the brain's autoregulatory capacity.

Our case highlights the importance of recognizing PRES as a potential manifestation of autonomic dysfunction in MSA, even in the absence of clinically significant orthostatic hypotension. It is important to highlight that, even when clinically silent, autonomic failure can be prognostically significant and cause significant secondary pathology.^4^ Continuous blood‐pressure monitoring and careful management strategies are essential, to mitigate extreme fluctuations. Strategies such as nocturnal antihypertensive therapy and daytime volume expansion may help to balance the risks of both supine hypertension and orthostatic hypotension.^6^

This case underscores the need for further research into the cerebrovascular consequences of autonomic failure in neurodegenerative disorders. It also builds on existing cases in the literature, to suggest that autonomic instability may be a risk factor for PRES, expanding the recognized spectrum of predisposing conditions to this syndrome.^1^, ^2^, ^3^, ^9^, ^10^ Clinicians should maintain a high index of suspicion for PRES in MSA patients presenting with acute neurological symptoms, particularly in those also on other potentially offending medications.

## Author Roles

(1) Research project: A. Manuscript: writing first draft; (2) Manuscript Preparation: A. Manuscript: review and critique.

J.M.: 1A.

J.L., Y.S., A.L.O.: 2A.

## Disclosures

**Ethical Compliance Statement:** An IRB was not required for this work. Written consent from the patient was obtained for publication of the case. We confirm that we have read the Journal's position on issues involved in ethical publication and affirm that this work is consistent with those guidelines.

**Financial Disclosures and Conflicts of Interest:** No specific funding was received for this work. The authors declare that there are no conflicts of interest relevant to this work.

**Financial Disclosures for the Previous 12 Months:** ALO receives funding from the Michael J. Fox Foundation, the American Parkinson Disease Association, and the Parkinson Study Group. JL is an expert respondent for the National Vaccine Injury Compensation Program. JM and YS have no financial disclosures.

## Financial Disclosures and Conflicts of Interest

Author disclosures are available in the Supporting Information.

## Supporting information


**Data S1.** Coi_disclosure.

## Data Availability

Data sharing not applicable to this article as no datasets were generated or analysed during the current study.
